# Case Report: Recurrent peptic ulcer perforations—a possible role of median arcuate ligament syndrome

**DOI:** 10.3389/fsurg.2026.1908665

**Published:** 2026-09-25

**Authors:** Mario Milotić, Luciana Milotić, Veronika Lekić Vitlov, Edo Bazdulj, Robert Radolović, Lada Kalagac Fabris, Slavica Kvolik

**Affiliations:** 1 Department of Surgery, Pula General Hospital, Pula, Croatia; 2 Department of Digestive Surgery, Clinical Hospital Centre Rijeka, Rijeka, Croatia; 3 Faculty of Medicine, University of Rijeka, Rijeka, Croatia; 4 Department of Anesthesiology, Resuscitation, and ICU, Pula General Hospital, Pula, Croatia; 5 Faculty of Medicine in Pula, Juraj Dobrila University of Pula, Pula, Croatia; 6 Department of Anesthesiology, Resuscitation, and ICU, Osijek University Hospital Center, Osijek, Croatia; 7 Faculty of Medicine, Josip Juraj Strossmayer University of Osijek, Osijek, Croatia

**Keywords:** abdominal pain, case report, celiac trunk, laparotomy, median arcuate ligament syndrome, peptic ulcer, truncal vagotomy

## Abstract

**Introduction:**

Median arcuate ligament syndrome (MALS), also known as Dunbar syndrome, is a rare vascular compression disorder caused by extrinsic compression of the celiac artery by the median arcuate ligament, affecting 2/100,000 people. Clinical presentation is often non-specific and may include postprandial abdominal pain, nausea, vomiting, diarrhea, and weight loss. Impaired splanchnic perfusion may contribute to gastrointestinal ischemia and, in rare cases, recurrent peptic ulcer disease. We report a patient with multiple episodes of perforated peptic ulcer disease associated with previously unrecognized MALS.

**Case presentation:**

A 65-year-old man underwent six emergency abdominal operations over an 11-year period for complications of recurrent peptic ulcer disease. His initial presentation in 2013 was with a perforated duodenal ulcer managed by Graham–Roscoe patch repair. Despite subsequent interventions, including repeated ulcer repairs and antrectomy with Billroth II reconstruction, the ulcer recurred. Investigations excluded *Helicobacter pylori* infection and Zollinger–Ellison syndrome. In September 2024, the patient presented with severe abdominal and thoracic pain. Multislice computed tomography angiography, performed to exclude acute aortic pathology, revealed significant compression of the celiac trunk, consistent with MALS. Surgical release of the median arcuate ligament combined with truncal vagotomy was performed. At 18-month follow-up, the patient remained free of abdominal pain, dyspeptic symptoms, and further ulcer-related complications.

**Conclusion:**

This case highlights MALS as a potential and often overlooked cause of refractory or recurrent peptic ulcer disease. In patients with repeated ulcer recurrence despite appropriate treatment and exclusion of common etiologies, vascular causes such as MALS should be considered. Early recognition may prevent repeated surgical interventions and improve long-term outcomes.

## 1 Introduction

Median arcuate ligament syndrome (MALS), also known as Dunbar syndrome, is a disorder caused by compression of the celiac trunk by the median arcuate ligament (MAL), characterized by weight loss, postprandial pain, nausea, and vomiting (1–3). Although celiac trunk stenosis is observed in one-third of autopsies, MALS is diagnosed in approximately 2/100,000 patients (1, 2). Diagnosis—in addition to medical history and clinical examination, during which a vascular bruit may be detected on auscultation of the epigastrium—is based on multislice computed tomography (MSCT) angiography and digital subtraction angiography (DSA) (4, 5). Peptic ulcer disease (PUD), on the other hand, has a considerably higher incidence of 1/1,000, with complications occurring in 0.7/1,000 patients (6, 7). Among the most common causes of peptic ulcer disease are *Helicobacter pylori* infection and nonsteroidal anti-inflammatory drugs (NSAID) overuse. A rarer cause is Zollinger–Ellison syndrome, which leads to hypersecretion of gastric acid (6). The primary mechanism of ulcer formation is believed to be a disruption of the balance between the damaging effect of gastric acid on the mucosa and mucosal defensive capacity; therefore, adequate perfusion of the gastric and duodenal mucosa is crucial in preventing peptic ulcer disease (7).

Although severe refractory ulcerations are typically attributed to uneradicated infection or acid hypersecretion, persistent mucosal hypoperfusion can critically disable local protective barriers, rendering the stomach highly vulnerable to recurrent ulceration and perforation—even under standard acid conditions or adequate antisecretory therapy (6, 7). Here, we present an 11-year clinical journey of a patient with multiple unexplained recurrent peptic ulcer perforations who was ultimately diagnosed with symptomatic MALS, highlighting the crucial and often overlooked role of celiac axis insufficiency in refractory PUD.

## 2 Case description

We present the case of a 65-year-old male patient (KH), with recurrent peptic ulcer perforations. The patient had no prior history of dyspepsia and presented first at the Emergency Department (ED) in October 2013 for intense, sudden gastric pain. The patient was otherwise healthy and denied use of NSAIDs or chronic therapy. He reported no allergies. He stated that he was an occasional alcohol consumer and a former smoker, having quit approximately 10 years ago. When he presented to the ED, he was hypotensive and tachycardic with signs of peritonitis. Radiographs of the abdomen showed free air, so the surgeon on call decided to perform an urgent exploratory laparotomy. During surgery, a perforated duodenal ulcer was found, and a Graham–Roscoe patch repair was performed. The patient was dismissed on the fifth postoperative day with standard instructions. Approximately 1 year later, in September 2014, the patient returned to the ED with clinical signs of acute abdomen, and another urgent surgical procedure was performed. A recurrent perforated duodenal ulcer was discovered, and a distal gastrectomy with Billroth II reconstruction was performed. The postoperative period was uneventful, and the patient was discharged on the seventh postoperative day with recommendations for antacid therapy. In June 2015, the patient underwent another urgent surgery for a perforation on the gastrojejunal anastomosis; sutures were performed.

During 2015–2016, Mr. KH was hospitalized twice for severe epigastric pain and was treated conservatively. The patient admitted to skipping the antacid therapy for a short period of a few days but resumed treatment as prescribed. In April 2016, the patient underwent another surgery for a perforation 2 cm from the gastroenteric anastomosis (GEA); sutures were performed. In August 2017, due to a new perforation, the gastroenteric anastomosis was taken down and reconstructed with a Roux-en-Y gastrojejunostomy.

In December 2018, the patient presented to the gastroenterology department with dyspeptic symptoms. He was treated conservatively, and an extensive work-up was performed, including *H. pylori* testing and a serum gastrin and secretin stimulation test, which excluded both Zollinger–Ellison syndrome and *H. pylori* infection. Esophagogastroduodenoscopy was performed, and a clean-based marginal ulcer was found on the gastrojejunostomy and treated according to guidelines (Figure 1).

**Figure 1:**
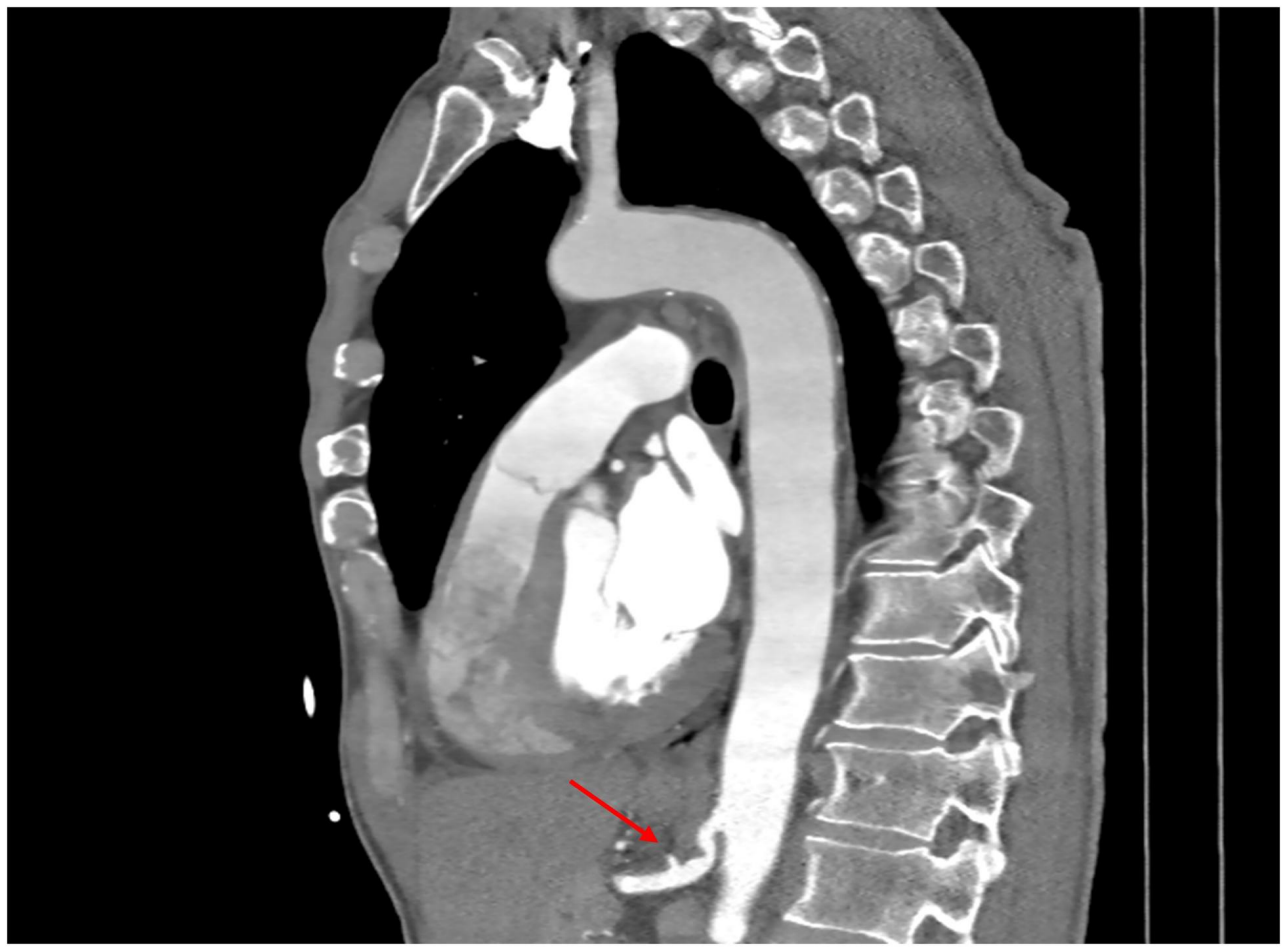
MSCT angiography:red arrow pointing celiac trunk narrowing due to MALS.

Finally, in September 2024, 11 years after the first symptoms, the patient presented to the surgery clinic with continuous intense pain in the left hemiabdomen and thorax. To exclude a dissecting aortic aneurysm, the patient underwent MSCT angiography, which revealed a 52% celiac trunk stenosis with post-stenotic dilatation of 6 mm and the characteristic hook sign on sagittal reconstructions—signs consistent with MALS (Figures 2–4). The patient’s celiac trunk exhibited marked tortuosity with S-shaped curving and exaggerated loops—rather than a direct linear trajectory toward its branches. This anatomical configuration increased flow resistance and could critically limit postprandial end-organ perfusion to the gastric mucosa (8).

**Figure 2:**
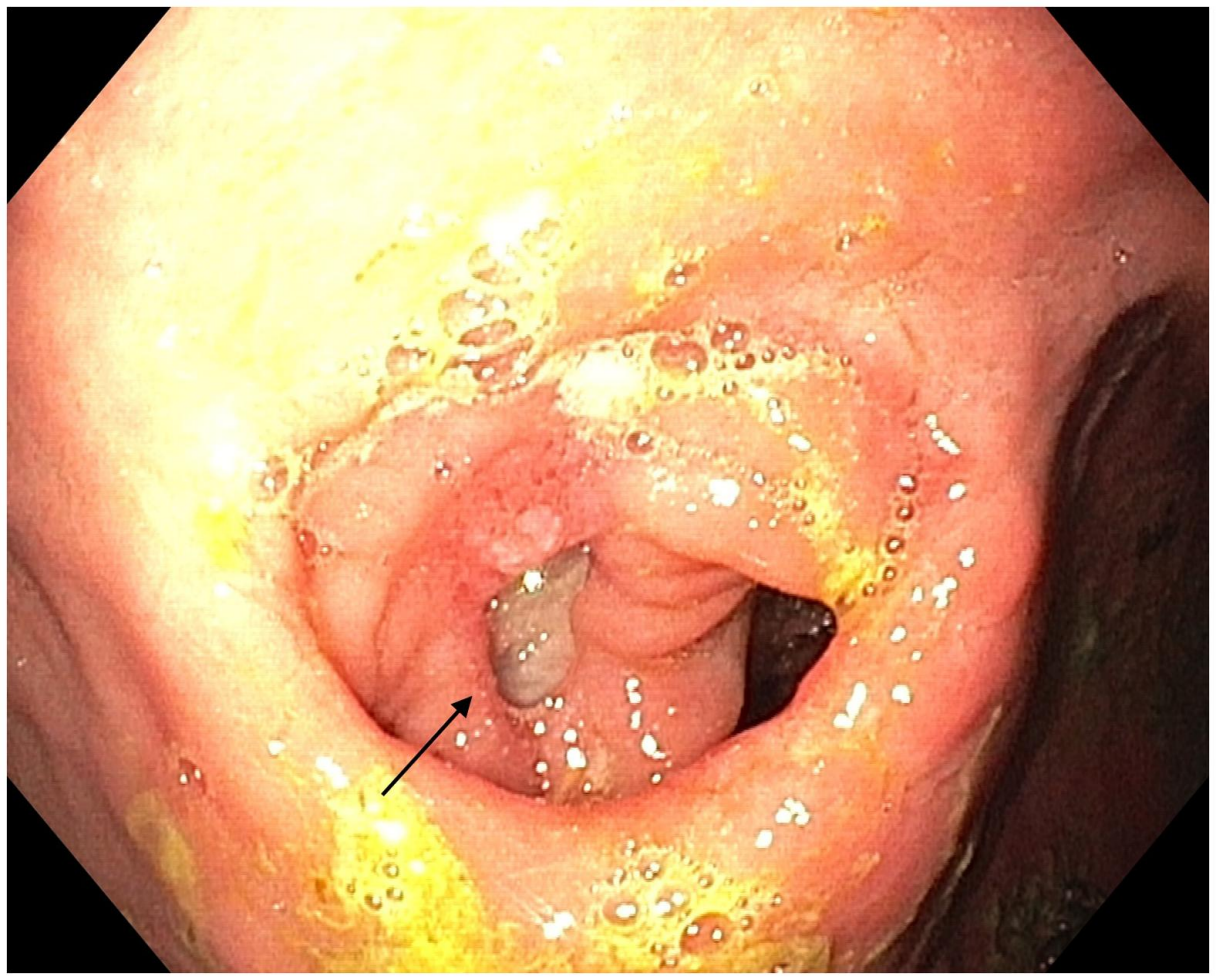
Esophagogastroduodenoscopy showing an ulceration on the gastrojejunostomy.

**Figure 3:**
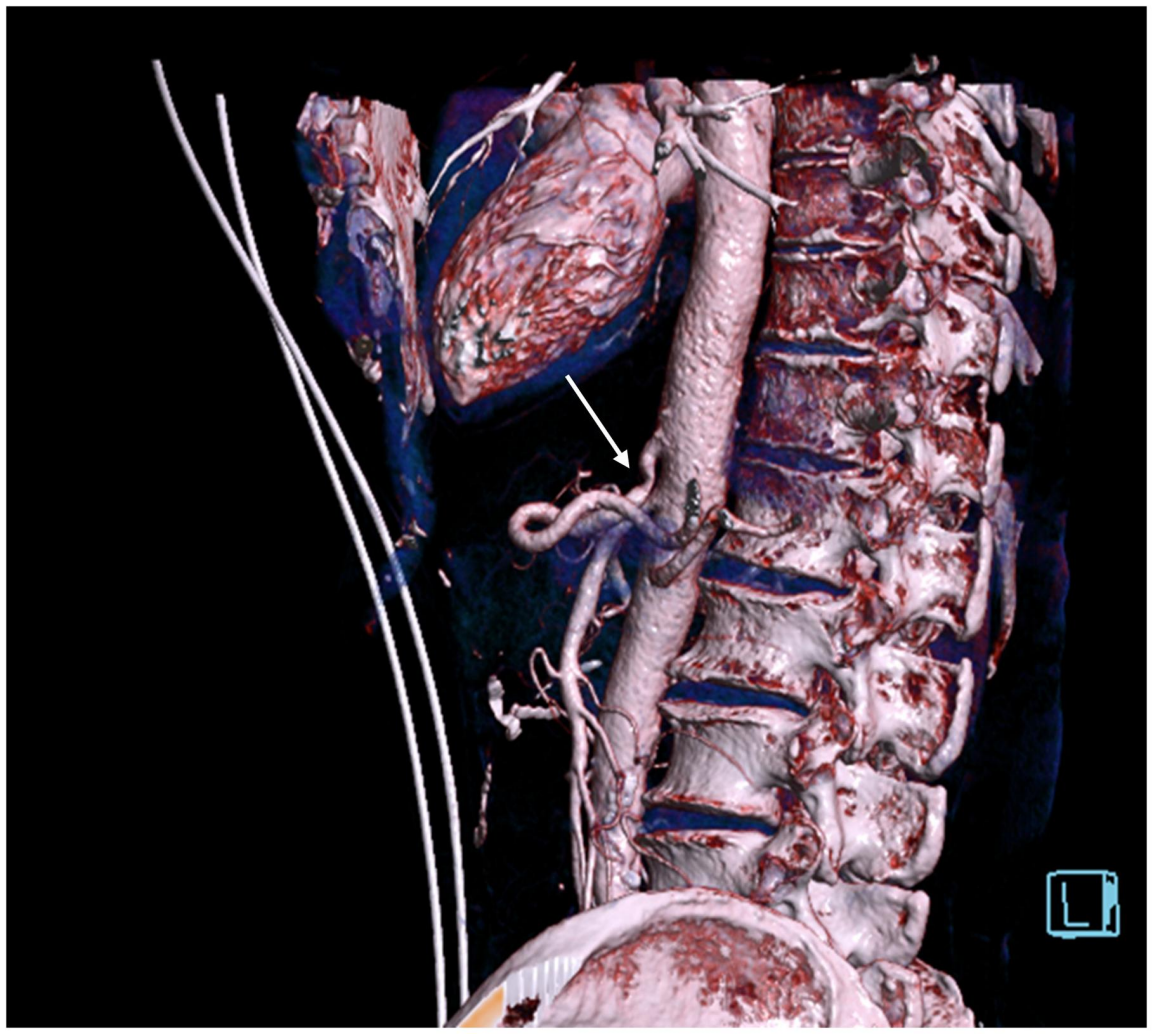
A 3D reconstruction of the MSCT angiography. The arrow highlights the narrowing of the celiac trunk caused by MALS.

**Figure 4:**
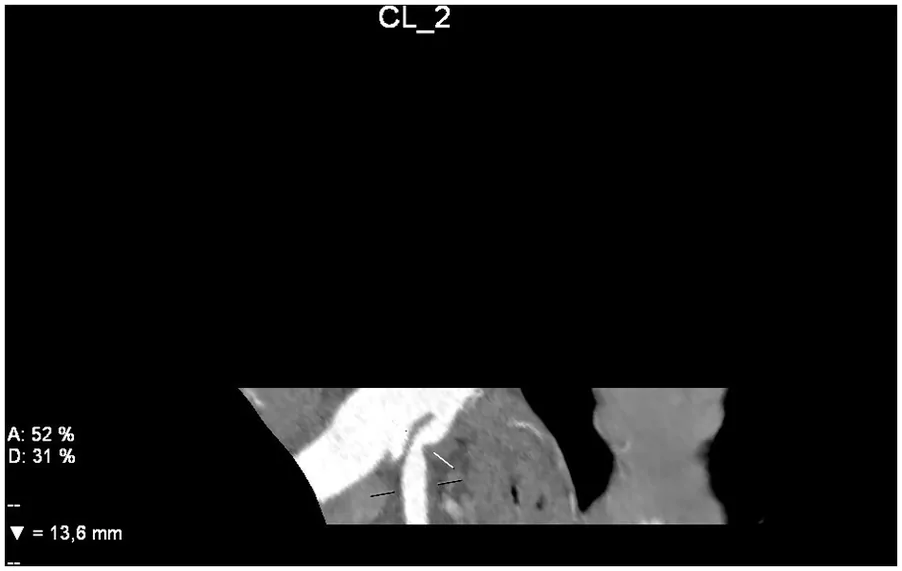
MSCT angiography scan showing the degree of celiac trunk stenosis.

The patient was prepared for surgery, and an open transection of the median arcuate ligament was performed. Intraoperatively, an ulcer was found on the gastrojejunostomy, which was perforated and adhered to the lateral abdominal wall, probably causing the pain in the left abdomen and thorax. Sutures of the ulcer were performed, as well as a truncal vagotomy. The operating surgeon did not obtain any biopsies of the ulceration since malignancy was not considered likely given the patient’s long history of benign ulcer perforation. The postoperative period was uneventful, and the patient was discharged on the fifth postoperative day.

## 3 Timeline

Table 1 presents the timeline of the clinical case, including all therapeutic and diagnostic procedures.

**Table 1:** Timeline of clinical symptoms and treatment in a 65-year-old male patient with recurrent peptic ulcer perforations and median arcuate ligament syndrome.

Date	Clinical episode	Intervention
October 2013	Presentation to ED with signs of acute abdomen, X-ray showing pneumoperitoneum	Emergency exploratory laparotomy and Graham–Roscoe repair; discharged on 5 POD
September 2014	Presentation to ED with signs of acute abdomen	Emergency explorative laparotomy showing perforated duodenal ulcer; antrectomy and Billroth II reconstruction performed; discharged on 7 POD
June 2015	Presentation to ED with acute abdomen, pneumoperitoneum	Emergency laparotomy: perforation of gastrojejunal anastomosis; sutures performed
2015–2016	Admitted to hospital twice due to abdominal pain	Conservative treatment
April 2016	Presentation with abdominal pain and distension	Emergency laparotomy: jejunal perforation at 2 cm from GEA; sutures performed
August 2017	Abdominal pain and distension	Emergency laparotomy, new perforation on GEA; takedown of GEA with Roux-en-Y reconstruction performed
2018–2019	Admission to gastroenterology department due to dyspeptic symptoms twice	Conservative treatment; tested for *H. pylori* infection negative, EGDS showing a clean-based marginal ulcer on the gastrojejunostomy—treated conservatively; Zollinger–Ellison syndrome excluded
September 2024	Acute left hemiabdomen and hemithorax pain; MSCT angiography performed to exclude aortic dissection	MSCT angiography shows MALS; surgery: transection of the median arcuate ligament and truncal vagotomy; discharged on 5 POD

POD, postoperative day; ED, emergency department; GEA, gastroenteric anastomosis; EGDS, esophagogastroduodenoscopy; MSCT, multislice computed tomography.

## 4 Patient perspective

The patient was contacted 18 months after the last surgery, in March 2026, and reported feeling well. He had experienced no abdominal pain or dyspeptic symptoms since the operation. Because of episodes of hypoglycemia, he was evaluated for late dumping syndrome and received dietary recommendations; however, as symptoms subsided, he did not complete the evaluation.

## 5 Discussion

This case has several interesting clinical implications. First, MALS is a rare condition, unfamiliar to many clinicians, that can cause non-specific gastrointestinal symptoms, making it hard to diagnose (1, 3). MALS may be the cause of peptic ulcer disease since it directly affects gastric and duodenal perfusion. It is well established that adequate perfusion is a key component of the mucosal acid barrier, and any disruption in the blood flow to the stomach and duodenum can predispose the mucous layer to injury (6, 7). That phenomenon is often seen in cases of hemodynamic shock with centralization of circulation, which is why clinicians often administer prophylactic proton pump inhibitors (PPI) to critical patients.

Second, recurrent peptic ulceration is usually caused by an underlying *H. pylori* infection or, in rarer cases, a gastrin-secreting tumor such as Zollinger–Ellison syndrome (6). Our patient underwent extensive work-up, which excluded those causes. The patient also denied abusing NSAIDs and admitted to only occasionally forgetting PPI therapy for a short period of time. Other causes of recurrent marginal ulcer perforation, including bile reflux, anastomotic ischemia, or residual acid secretion, were not assessed in this patient, which tempers causal attribution to MALS alone.

The definitive diagnosis of MALS was made only 11 years after the first presentation, and accidentally during evaluation of a different pathology, which emphasizes how vascular causes of peptic ulcer disease are rarely addressed. Although there is no definitive connection between MALS and peptic ulcer disease, the literature does hypothesize the role for chronic malperfusion in dyspepsia and peptic ulcer disease (1, 3).

The measured 52% stenosis is generally classified as moderate rather than severe; however, the clinical significance of celiac compression in MALS depends not only on the degree of luminal narrowing but also on the adequacy of collateral compensation. A limitation of our case is that the reported 52% stenosis was measured on a standard single-phase computed tomography angiography (CTA) acquisition, and the respiratory phase was not specified. Dynamic respiratory-phase imaging, which can influence the measured degree of celiac compression, was not performed. Nevertheless, van Petersen et al. demonstrated that patients with limited collateral mesenteric circulation are more likely to benefit symptomatically from median arcuate ligament release than those with extensive collateralization, implying that even moderate stenosis can become hemodynamically significant once compensatory pathways are compromised (9). In our patient, we hypothesize that the combination of moderate stenosis and disrupted collateral perfusion accounts for the clinical significance of MALS in this case.

Surgical treatment for MALS includes the transection of the median arcuate ligament, usually using a laparoscopic approach (2, 5, 10). In this case, however, an open approach without previous exploratory laparoscopy was chosen. This decision was made preoperatively because all previous operative reports described multiple strong adhesions. Intraoperatively, dense adhesions involving the transverse mesocolon, the gastric remnant, and the anterior abdominal wall were found, and a meticulous adhesiolysis was performed, combining sharp dissection and a bipolar device. When the median arcuate ligament compressing the celiac trunk was finally identified, it was sharply transected under direct visualization, and completeness of release was assessed by inspection and palpation. Unfortunately, no postoperative vascular imaging was performed, which is another limitation of this case. However, the patient’s sustained symptom-free course is encouraging and supports the association between MALS and recurrence of peptic ulcer perforation.

During surgery, a truncal vagotomy was performed in this patient to reduce acid secretion. This decision was made intraoperatively upon identification of the perforated ulcer adherent to the lateral abdominal wall. The operating surgeon, considering the patient’s history and extensive work-up, decided to combine the acid suppression from vagotomy with improved perfusion from release of the median arcuate ligament to minimize the possibility of recurrence. This decision, however, is the most significant limitation in attributing the favorable outcome solely to release of the median arcuate ligament, since truncal vagotomy is a well-established treatment for refractory peptic ulcer disease. To investigate the role of MALS in recurrent ulcer perforations, a staged approach—including release of the median arcuate ligament and eventual truncal vagotomy in case of recurrence—would be preferred; however, this was deemed not feasible or ethical in this patient given the already high number of previous surgeries. However, it must be considered that the patient was, for the vast majority of the time (excluding self-reported lapses in adherence, which were short and unrelated to the hospitalizations), receiving PPI therapy, which achieves acid suppression at least comparable to truncal vagotomy and has a low unresponsiveness rate of 10%–15% (11). This raises the possibility that acid hypersecretion was not the primary cause of recurrence in this patient and that impaired mucosal defense due to chronic hypoperfusion may have had an important role. Therefore, we think that a key indicator of successful MAL release in this patient, alongside restored vascular perfusion, was the complete resolution of the refractory chronic abdominal pain that had previously required two hospitalizations. Crucially, although the patient also underwent a truncal vagotomy, this procedure does not alleviate MALS-induced neurogenic pain. Rather, pain relief in MALS relies on surgical decompression of the ligament itself, often augmented by celiac ganglionectomy to directly disrupt chronic pain signaling (12).

Furthermore, this case underscores the importance of considering vascular causes in cases of refractory peptic ulcer disease and chronic abdominal pain, especially when classic and common causes have been excluded. It can be argued that the duodenum and stomach have a rich collateral network, mostly through the gastroduodenal (GDA) and pancreaticoduodenal (PDA) arteries, which should be sufficient to maintain adequate perfusion of the above-mentioned organs, even in cases of MALS (13). However, it must be considered that our patient had undergone multiple abdominal surgeries and a gastric resection, which surely affected the collateral network. In addition, collateral flow through the GDA and PDA occurs in reverse due to a change in the pressure gradient caused by MALS (13). This flow has a lower perfusion pressure that is probably sufficient for macroscopic perfusion during fasting periods, but during postprandial periods, it causes relative mucosal ischemia, which can lead to an imbalance between the mucosa’s protective mechanisms and the corrosive action of gastric acid.

An important clarification is that the patient's collateral mesenteric flow through the GDA and PDA was presumably intact at the time of the first two perforations in 2013 and 2014. The patient had not undergone gastric resection at that time. This suggests that the initial ulcers more likely represented standard, multifactorial peptic ulcer disease, occurring independently of any collateral flow compromise. We hypothesize that MALS-related hypoperfusion became a clinically relevant contributor only after the distal gastrectomy with Billroth II reconstruction performed in September 2014. In addition to median arcuate ligament compression, post-stenotic celiac trunk tortuosity (Figure 1) likely contributed to hypoperfusion. The increased vessel length, narrowed radius, and sharp curvature elevate hemodynamic resistance, causing a localized pressure drop that ultimately impairs end-organ perfusion (8).

An additional limitation is the absence of histopathological confirmation of a benign ischemic ulcer base. Recurrent marginal ulceration in a 65-year-old patient warrants formal exclusion of malignancy (e.g., adenocarcinoma or marginal zone lymphoma) regardless of clinical suspicion, which was not performed in our case.

## 6 Conclusion

MALS is a rare condition and is challenging to diagnose since it produces no specific symptoms. Although it is impossible to establish a causal relationship between MALS and recurrent peptic ulcer perforation from a single case, this report highlights MALS as a possible under-recognized factor. Therefore, when all the typical causes of recurrent peptic ulcer disease are excluded, clinicians should suspect and test for MALS. Adequate radiologic evaluation can spare patients unnecessary diagnostic and treatment delays.

## Data Availability

The raw data supporting the conclusions of this article will be made available by the authors without undue reservation.
